# Identification of a postnatal period of interdependent neurogenesis and apoptosis in peripheral neurons

**DOI:** 10.1242/bio.060541

**Published:** 2024-11-11

**Authors:** Catherine L. Kaminski, Debarghya Dutta Banik, Ligia B. Schmitd, Brian A. Pierchala

**Affiliations:** ^1^ Department of Anatomy, Cell Biology & Physiology, Stark Neurosciences Research Institute, Indiana University School of Medicine, Indianapolis, IN 46202, USA; ^2^ Department of Biologic and Materials Sciences, University of Michigan, Ann Arbor, MI 48109, USA

**Keywords:** Apoptosis, Neurogenesis, Superior cervical ganglion, Geniculate ganglion, *Bax*, Programmed cell death, Peripheral neuron, Development

## Abstract

During neurogenesis, excessive numbers of neurons are produced in most regions of the central and peripheral nervous systems. Nonessential neurons are eliminated by apoptosis, or programmed cell death. This has been most thoroughly characterized in the peripheral nervous system (PNS) where targets of innervation play a key role in this process. As maturing neurons project axons towards their targets of innervation, they become dependent upon these targets for survival. Survival factors, also called neurotrophic factors, are produced by targets, inhibit apoptosis cascades, and promote further growth and differentiation. Because neurotrophic factors are limited, as is target size, neurons that do not correctly and efficiently innervate targets undergo apoptosis ( Levi-Montalcini, 1987; Davies, 1996). Thus, excessive neurogenesis acts to ensure that sufficient numbers of neurons are produced during development. In the superior cervical ganglion (SCG), this process of neurogenesis and subsequent apoptosis is reported to be complete by postnatal day 3-4 (P3-P4) in mice. Surprisingly, we observed significant numbers of apoptotic neurons out to P14, and neurogenesis was still present at P14 as well. In both the SCG and geniculate ganglion (GG), postnatal neurogenesis was dependent on apoptosis because little or no postnatal neurogenesis was observed in *Bax*^-/-^ mice, in which apoptosis is eliminated. These results indicate that both neurogenesis and apoptosis continue to occur well after birth in peripheral ganglia, and that neurogenesis depends on apoptosis, suggesting that neurogenesis continues postnatally to replace neurons that are eliminated during synaptic refinement.

## Teaser

**Summary:** Neurogenesis and subsequent apoptosis are thought to conclude perinatally in the PNS. This paper demonstrates that both neurogenesis and apoptosis continue at low levels for weeks after birth. Surprisingly, neurogenesis depends on apoptosis, suggesting that some neurons eliminated during synaptic refinement are replaced.

## INTRODUCTION

During development of the nervous system, most populations of neurons require neurotrophic factors supplied by targets of innervation, assuring proper target engagement. While neurotrophic factors ensure survival of innervating neurons, competition pathways actively promote apoptosis of neurons that are not receiving sufficient survival signals, narrowing the developmental time period of programmed cell death to several days (Deppmann et al., 2008; Singh et al., 2008). In peripheral ganglia such as the superior cervical ganglia (SCG), dorsal root ganglia (DRG) and geniculate ganglia (GG), neurogenesis occurs predominantly prenatally in mice and rats and is thought to be complete postnatal day 3-4 (Holt et al., 2021; Mendez-Maldonado et al., 2020). Programmed cell death occurs several days later, perinatally, and is also reported to be complete within the first postnatal week.

Nearly all studies that examined developmental apoptosis of PNS ganglia used total cell counts to monitor the loss of neurons. Similarly, proliferation in PNS ganglia has been determined predominantly with earlier, less sensitive methods such as tritiated-thymidine labeling of dividing cells (Hendry, 1977; Wright et al., 1983; Brennan et al., 1999; Bertrand et al., 2008; Fagan et al., 1996). Analysis of total neuron counts has the potential to be misleading if neurogenesis is occurring at the same time that apoptosis is occurring. For this reason we evaluated programmed cell death using cleaved caspase-3 labeling, a faithful marker of the terminal stage of apoptosis in the SCG (Wright et al., 2007; Deshmukh et al., 1996; Kristiansen and Ham, 2014). We found that apoptosis occurred at a low level for more than 2 weeks after birth and did not end perinatally. To examine neurogenesis, we utilized EdU labeling that detects cell division while more easily allowing for co-immunolabeling. This analysis revealed a low level of neurogenesis that also occurred well into postnatal age in both the SCG and the GG, indicating postnatal neurogenesis is common to PNS populations that are derived from both the neural crest and neurogenic placodes. Importantly, both apoptosis and neurogenesis were eliminated in *Bax*^-/-^ mice, suggesting that neurogenesis requires apoptosis and that these two events are linked.

## RESULTS

### Programmed cell death in the SCG continues well into postnatal age

**Fig. 1.**
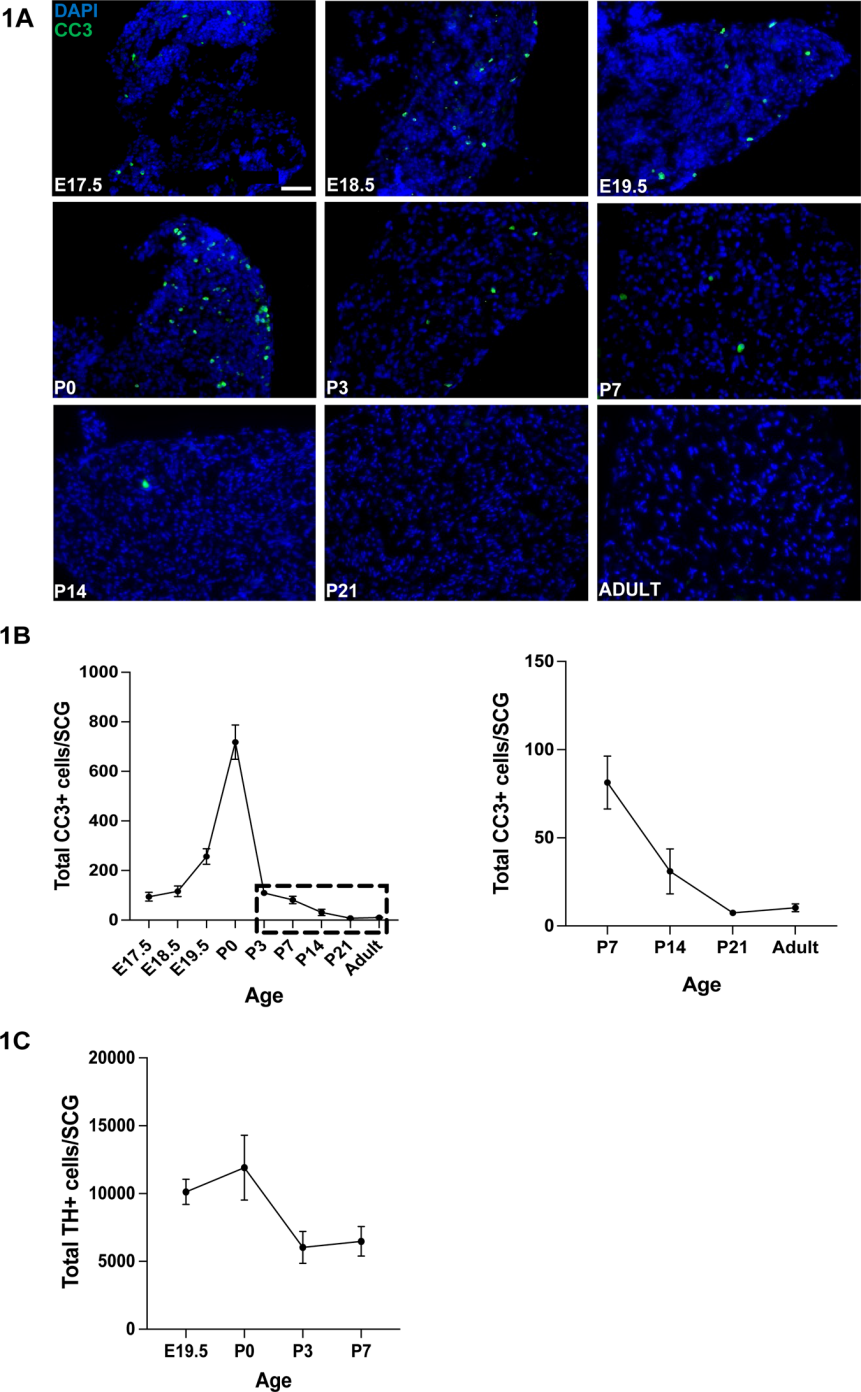
**Programmed cell death in the SCG continues into postnatal age.** (A) Sections of SCGs from mice that were E17.5 to adulthood (P60) were immunolabeled for cleaved caspase-3 (CC3, green) and nuclei (DAPI, blue). Scale bar: 50 µm. (B) Quantification of the number of CC3+ neurons per ganglion reveals that the number of apoptotic cells in the SCG peaks at P0 and decreases thereafter. The left graph shows the entire time-course and the right graph shows only P7 to adulthood. (C) Total neuron counts, as ascertained by counting all TH+ cells per ganglion, was quantified from E19.5 to P7. Error bars for all graphs are mean±s.e.m., and *n*=3 for all ages.

To identify the entire developmental period of apoptosis and neurogenesis, we used more sensitive methods, cleaved-caspase-3 immunolabeling and 5-ethynyl-2′-deoxyuridine (EdU) labeling, respectively. Apoptosis has been thoroughly examined in the SCG, which has long been an eminent model for studies of programmed cell death. While apoptosis was reported to cease by P3-P4 in mice (Brennan et al., 1999; Bertrand et al., 2008; Fagan et al., 1996; Deppmann et al., 2008), we examined apoptosis over a larger range of postnatal ages. SCGs were dissected from mice at E17.5 through adulthood, serially sectioned and immunolabeled for cleaved caspase-3 (CC3). As expected, there was a peak of apoptotic cells at P0, and cell death rapidly declined thereafter (Fig. 1A,B). There were apoptotic neurons detected as early as E17.5, but at considerably lower levels than at P0. Interestingly, although the number of apoptotic cells present in the SCG at postnatal ages after P0 were lower, CC3+ neurons were present at P3, P7 and P14, slowly declining to a minimum at P21 (Fig. 1A,B). The steady decline in the number of apoptotic neurons from P3-P21 suggests that this is not simply a low, basal level of CC3 staining (Fig. 1B). To confirm that CC3+ cells were neurons, and not glial cells, tyrosine hydroxylase (TH) was immunolabeled at the same time and only cells that were both TH+ and CC3+ were reported as apoptotic neurons (Fig. 1A,B). Because apoptosis in the SCG has not been reported at these later ages, we also evaluated cell death by counting the total number of TH+ neurons in the SCG. Consistent with what has been reported previously (Deppmann et al., 2008; Brennan et al., 1999; Bertrand et al., 2008), the number of neurons in the SCG drops by almost 50% between P0 and P3, and there was no further change detected at P7 by measuring total neuron numbers (Fig. 1C).

### Postnatal apoptosis in the SCG is BAX-dependent

**Fig. 2.**
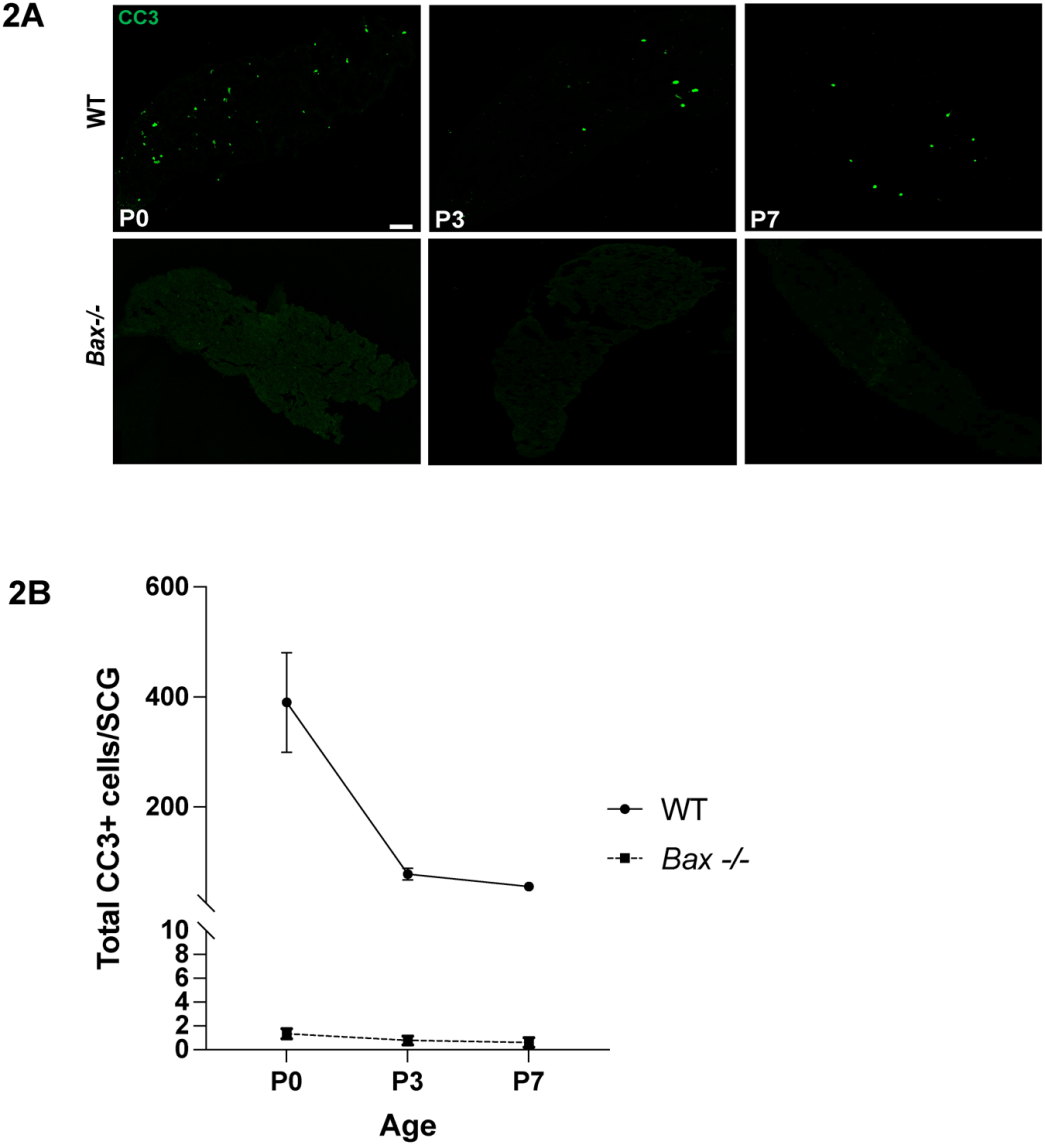
**Postnatal apoptosis in the SCG is BAX-dependent.** (A) SCGs from P0, P3 and P7 mice were immunolabeled for CC3 in green. The SCGs were from wild-type (WT) animals (*Bax*^+/+^ or *Bax*^+/-^, top row) and *Bax*^-/-^ mice (bottom row). Scale bar: 75 µm. (B) Quantification of the number of CC3+ cells per ganglion in *Bax*^+/+^ and *Bax*^+/-^ mice (WT, solid line) and *Bax*^-/-^ mice (dashed line) are shown at ages P0-P7, revealing a dramatic loss of apoptosis at all ages. Mean±s.e.m. is displayed for all quantifications, and *n*=3-6 for all ages and genotypes.

Sympathetic neurons require NGF for survival during development, and competition for target-derived NGF accounts for programmed cell death observed perinatally (Levi-Montalcini, 1987; Davies, 1996). NGF withdrawal triggers the intrinsic apoptosis pathway that leads to the activation of caspase-9, which in turn activates the terminal caspase-3 (Deshmukh et al., 1996; Li et al., 1997; Zou et al., 1997; Hao et al., 2005; Wright et al., 2007). The pro-apoptotic *Bcl-2* family member *Bax* is required for this intrinsic pathway of cell death in SCG neurons, and *Bax*^-/-^ mice have dramatic reductions in apoptosis in the SCG and other peripheral ganglia (Deckwerth et al., 1996; Middleton and Davies, 2001; White et al., 1998). To determine whether SCGs from *Bax^-/-^* mice had apoptosis in postnatal ages, we examined SCGs at P0, P3 and P7 in *Bax^+/+^* and *Bax^-/-^* mice. CC3 labeling revealed that there was no apoptosis observed at any age in *Bax^-/-^* SCGs as compared to *Bax^+/+^* SCGs (Fig. 2A,B), demonstrating that *Bax* is obligate for cell death at any age in the SCG.

### Neurogenesis occurs postnatally in peripheral ganglia

**Fig. 3.**
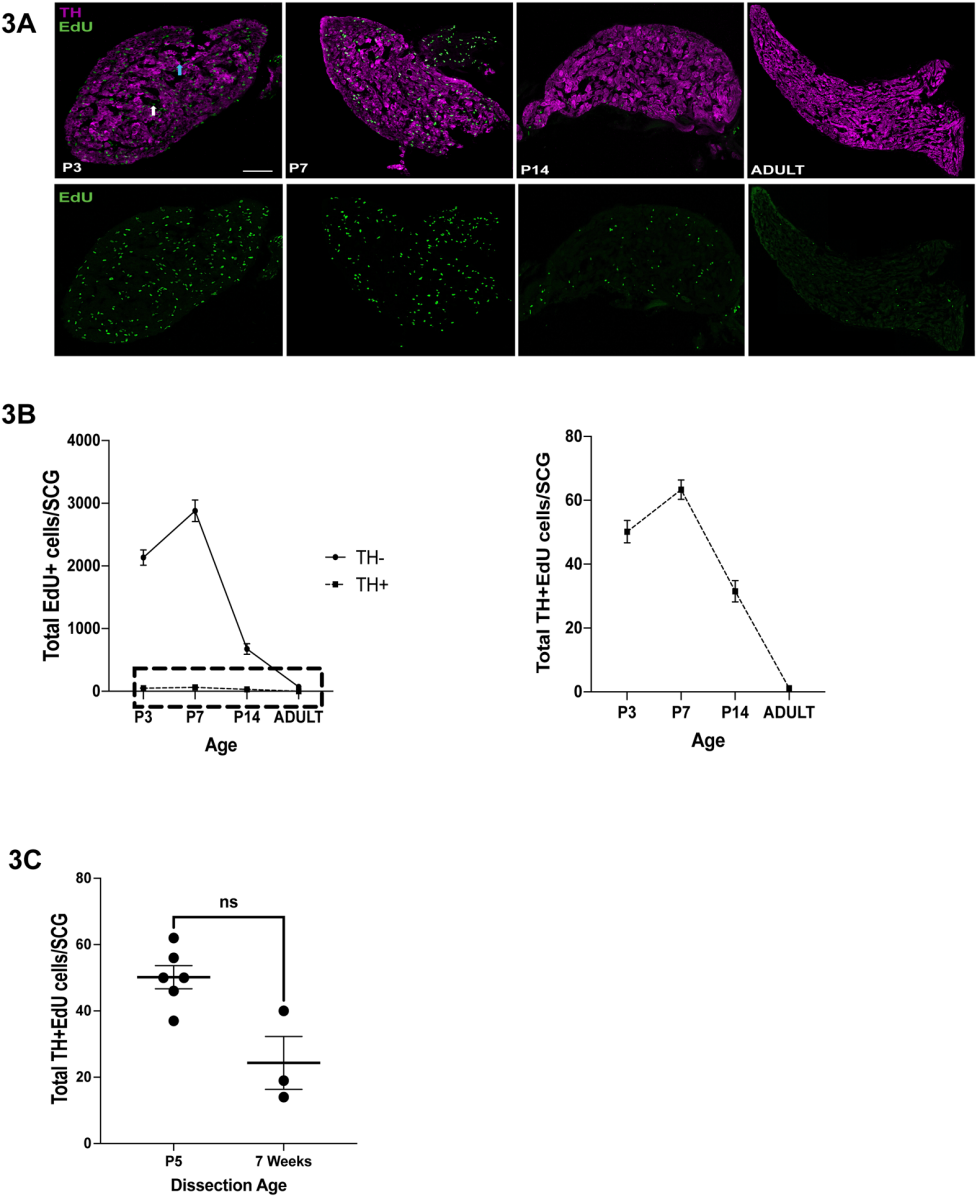
**Neurogenesis occurs postnatally in the SCG.** (A) Sections of SCGs were labelled with EdU (green) and immunolabeled for TH (magenta) from mice of ages P3 to adult. Mice were injected with EdU at the age indicated in each panel and the SCGs were dissected 24 h later. Scale bar: 75 µm. (B) EdU+ cells were quantified and determined to be either TH+ (neurons) or TH- (glia), and these values were graphed as EdU+/TH- cells (solid line) or EdU+/TH+ cells (dashed line). The right graph shows only EdU+/TH+ neurons. (C) Mice were injected with EdU at P3 and the SCGs were isolated from the mice at P5 or 7 weeks of age and analyzed as in B. Data are represented as mean±s.e.m. in all panels; *n*=3-8 mice for each age and condition.

**Fig. 4.**
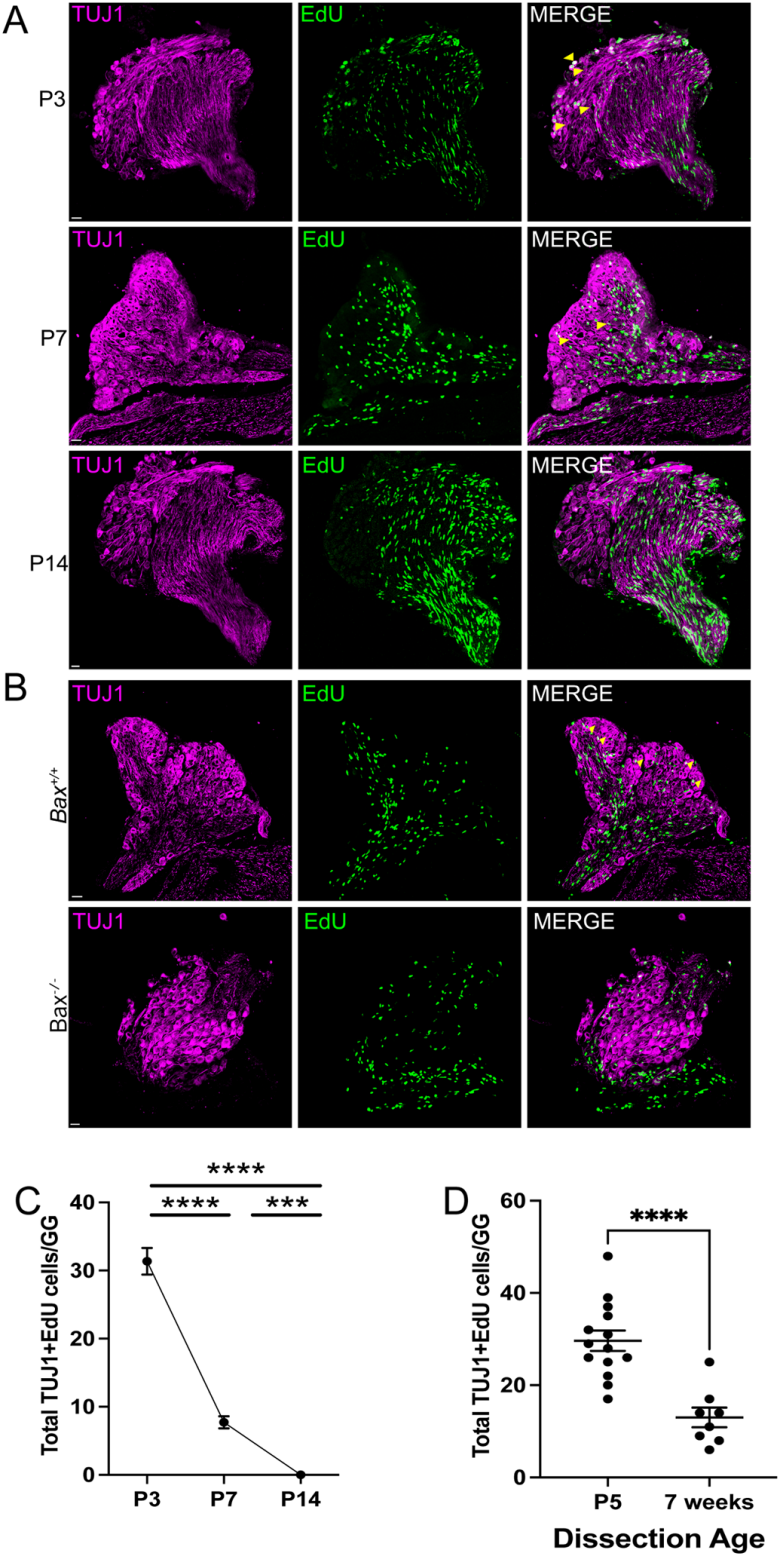
**Neurogenesis occurs postnatally in the GG.** Mice were injected with EdU at P3, P7 or P14, as was done for analysis of the SCG, and the GG were collected 48 h later. EdU labelling was performed in combination with TUJ1 labeling to identify neuronal cell bodies (A). (B) Either *Bax*^+/+^ and *Bax*^-/-^ mice were injected with EdU at P3, the ganglia collected 48 h later, and then analyzed as in A. Yellow arrowheads in the merged images indicate newly-born neurons that are both EDU+ and TUJ1+. (C) The number of EdU+ sensory neurons (TUJ+) in the GG were quantified at P3, P7 and P14. (D) P3 mice were injected with EdU and the GGs isolated 2 days or 3-6 weeks later, and then analyzed as in A and B. Data are represented as mean±s.e.m. in all panels; *n*=3-8 mice for each age and condition.

Neurogenesis in the SCG has been reported to occur in mice and rats prenatally, rapidly declining after birth (Fagan et al., 1996; Hendry, 1977; Mendez-Maldonado et al., 2020). Because these studies were conducted using histologic methods that do not allow dual labeling to identify which type of cell was generated (neurons or glia), we utilized EdU labeling of dividing cells. To this end, postnatal mice ranging from P3 to adulthood were given a single injection of EdU (8 µg/gm bodyweight), and 48 h later the SCGs collected, serially sectioned, and labelled for EdU and TH. The vast majority of dividing cells in the SCG at postnatal ages did not express TH and had elongated, bean-shaped nuclei, indicating they were glia (Fig. 3A,B). There were, however, EdU+ cells that also expressed TH, indicating the presence of newborn neurons. Newborn neurons were observed at P3, P7 and P14, and neurogenesis was not observed in SCGs from adult mice (P60, Fig. 3B). This surprising observation raised the question of whether postnatal neurogenesis occurs in other peripheral ganglia, or whether it is unique to sympathetic neurons. Neurogenesis and apoptosis in the GG occurs earlier than in the SCG, which is common for craniofacial ganglia that are derived from neurogenic placodes (Stark, 2014; Quina et al., 2012). Neurogenesis in the GG peaks at E10.5-11.5 in mice and declines thereafter, and is thought to be finished well before birth (Altman and Bayer, 1982). Programmed cell death in the GG occurs in two waves, an early peak at E11.5 and a later peak at E14.5 (Conover et al., 1995; Liu et al., 1995; Patel and Krimm, 2012). We injected mice with EdU at P3, P7 or P14 and examined neurogenesis in GGs in the same manner that was done for the SCG. Interestingly, EdU+ neurons were observed at P3 and P7, but were not observed at P14, indicating that postnatal neurogenesis ends earlier in the GG than in the SCG (Fig. 4A,C). EdU+ neurons were also β-Tubulin+, indicating they are neurons that innervate the oral cavity or pinna, the two targets of the GG (Fig. 4A,C).

While the number of neurons that are born postnatally in the SCG from P3-P14 may seem low, EdU is unstable and rapidly degrades upon injection, thus only labeling dividing cells for 6-12 h. Therefore, if twice this number are born every day from P3-P14, this late born population is between 770-1320 neurons, which is a significant percentage of the SCG, given it only comprises between 6000-10,000 neurons in mice (Fig. 1C). Likewise, the numbers of GG neurons born between P3-P7 are between 48-120 neurons in just this 4-day postnatal period. Because there are only 1000-1200 sensory neurons in each GG, postnatal neurogenesis produces a significant percentage of the population, between 5-10% as a conservative estimation.

In other locations in the nervous system, newborn neurons often rapidly undergo apoptosis. To determine whether these postnatally-born neurons persist for longer than 48 h, P3 mice were injected once with EdU, and the SCGs and GGs were collected either 2 days later, as before, or 3-7 weeks later. There was an approximately 50% decline in the number of EdU+ neurons remaining 3-7 weeks post injection (Figs 3C, 4D). These data indicate that a significant portion of these neurons either underwent apoptosis and were lost, or continued to divide, thereby diluting the EdU label and causing them disappear over continued cell divisions.

### Postnatal neurogenesis depends on apoptosis

**Fig. 5.**
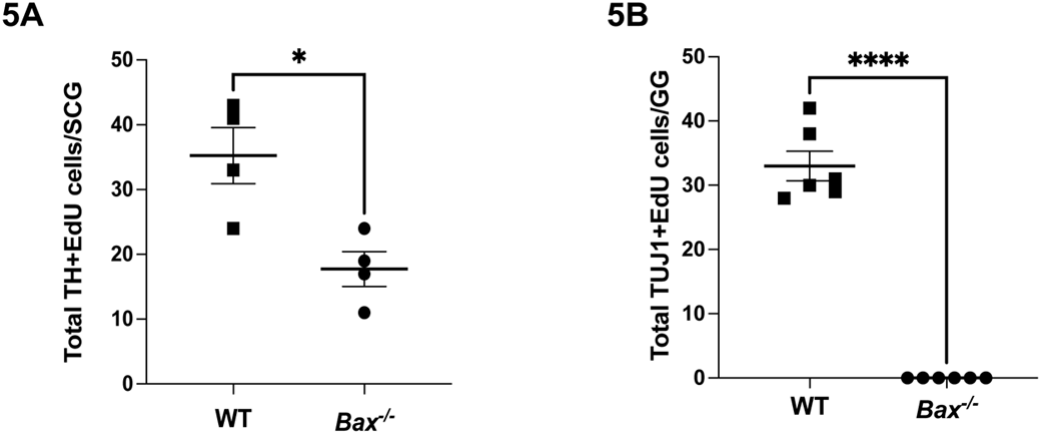
**Postnatal neurogenesis depends on apoptosis.** P3 *Bax*^+/+^ (WT) and *Bax*^-/-^ mice were injected with EdU and 48 h later the SCG and GG were isolated. EdU labeling was imaged from sections of these ganglia and quantified in combination with TH for SCG neurons (panel A) and TUJ1 for GG neurons (panel B). Data are mean±s.e.m. and *n*=4-6 mice were analyzed of each genotype. Quite remarkably, both in the SCG and GG, there was a significant loss of EdU+ neurons in *Bax*^-/-^ mice as compared to *Bax*^+/+^ mice, indicating that postnatal neurogenesis depends on neuronal apoptosis.

While neurogenesis has been thought to generally precede programmed cell death, this postnatal neurogenesis in the SCG is occurring simultaneously when other neurons are undergoing apoptosis. This raises the possibility that this late wave of neurogenesis may be dependent on, or related to, developmental apoptosis. To evaluate this, *Bax^-/-^* mice were used to eliminate all apoptosis in the SCG. *Bax^-/-^* and *Bax^+/+^* mice were injected with EdU at P3, and after 48 h the SCGs and GGs were isolated and neurogenesis was evaluated as before. Surprisingly, in both SCG and GG, there was a dramatic decline in the number of EdU+ neurons (colabelled with TH or TUJ1, Fig. 5). In the GG all postnatal neurogenesis was eliminated in *Bax^-/-^* mice, and greater than 50% was lost in the SCG, indicating that postnatal neurogenesis depends on apoptosis.

While essentially all SCG neurons are noradrenergic and express TH, recent single cell RNA sequencing studies of sympathetic ganglia suggest that there are at least five distinct subpopulations of noradrenergic neurons and two populations of cholinergic neurons (Zeisel et al., 2018; Mapps et al., 2022; Furlan et al., 2016). Their specific physiologic function and targets of innervation not fully resolved. To determine whether late-born neurons may represent a specific subpopulation that is produced postnatally, we examined whether EdU+ neurons expressed either RARRES or NPY, markers of two SCG noradrenergic subpopulations. Immunolabeling for NPY and RARRES revealed subsets of neurons that expressed either marker, although expression increased postnatally, and immunolabelling best labelled neurons at P14 (Fig. S1). When co-labelling studies were performed, EdU+ cells that appeared postnatally did not express RARRES or NPY (Fig. S1). In fact, neither RARRES+ nor NPY+ cells underwent postnatal apoptosis either (Fig. S1), indicating these populations are born and undergo programmed cell death prior to P3.

## DISCUSSION

The data presented here reveal a new period of neurogenesis and apoptosis in the SCG, from P3 to P21. Unlike programmed cell death during perinatal age, which is a rapid peak followed by a rapid decline of apoptosis between E18.5 and P3, postnatal cell death occurs at a low level over the course of three weeks. Likewise, postnatal neurogenesis occurred at a low rate in both the SCG and GG over the course of days to weeks at the same age period, and likely accounts for between 5-20% of the total neuronal population in these ganglia. Importantly, postnatal neurogenesis depended upon apoptosis in these ganglia, suggesting that neurons produced postnatally are in response to an “excessive” amount of apoptosis, perhaps fine-tuning the final number of neurons necessary for circuit function. This fundamentally changes the model of development of peripheral ganglia from neurogenesis preceding cell death, and apoptosis due to neurotrophic factor competition, to a model that now includes a prolonged postnatal period of interdependent neurogenesis and cell death that adjusts the final number of neurons.

These provocative data raise several questions. In the SCG, for example, what subpopulation of sympathetic neurons are these late born cells? Do they contribute to a specific subpopulation other than RARRES+ and NPY+ neurons, or do they contribute to several different subpopulations equally? Do these neurons project to a specific target, or set of targets? One possibility is that these neurons project to the targets farthest from the SCG, such as the pineal gland or iris, which accounts for their later development at postnatal age. It is also tempting to speculate that postnatal apoptosis represents the elimination of some of these postnatal-born neurons that may be produced in excess. Indeed, some of the EdU+ neurons appeared to be undergoing apoptosis, consistent with this notion. Likewise, we found that as much as 50% of the number of neurons labeled with EDU one day after injection were lost within a few weeks, possibly due to apoptosis.

The dependence of postnatal neurogenesis on apoptosis raises interesting mechanistic questions. Presumably some aspect of the programmed cell death pathway is being “sensed” by stem cells in the ganglia, leading to heightened neurogenesis. This would explain why postnatal neurogenesis ceases around the same developmental age that PCD does. A direct demonstration of whether apoptosis of SCG neurons modulates the level of postnatal neurogenesis would be to increase the amount of cell death, perhaps by blocking NGF with injections of function blocking antibodies, or by eliminating neurons with sympathetic-selective toxins such as guanethidine or 6-hydroxy dopamine. When adult rats are administered guanethidine, resulting in the elimination of 50% of SCG neurons, there was no recovery in the number of TH+ neurons in the ganglion 3-6 months later, indicating that the adult SCG is no longer capable of responding to the loss of SCG neurons via neurogenesis (Walters et al., 2020). This suggests that the process of apoptosis and neurogenesis in early postnatal mice is likely to be a purely developmental event. Finally, because postnatal neurogenesis was observed in both SCG and GG, it is possible that this is a common phenomenon among neural crest-derived and placode-derived peripheral ganglia, suggesting that postnatal neurogenesis contributes to sensory, autonomic and perhaps even motor circuits.

## MATERIALS AND METHODS

### Materials availability

All materials and software are commercially available, as described herein. For access to data or images please email the corresponding author.

### Animals

Experiments were conducted in compliance with the American Association for Accreditation of Laboratory Animal Care (AAALAC) and the Institutional Animal Care and Use Committee (IACUC) of Indiana University School of Medicine. For the developmental cell death experiments, C57BL/6J (Jackson Laboratories, Stock #000664) mice were used for all ages. The ages used were embryonic day 17.5 (E17.5), embryonic day 18.5 (E18.5), embryonic day 19.5 (E19.5), postnatal day 0 (P0), postnatal day 3 (P3), postnatal day 7 (P7), postnatal day 14 (P14), postnatal day 21 (P21) and adult (P60). For the postnatal apoptosis experiments both *Bax^-/-^* and WT (*Bax^+/-^ or Bax^+/+^*) (Jackson Laboratories, Stock #002994) mice were used at P0, P3 and P7. The production of *Bax*^-/-^ mice was accomplished by breeding *Bax^+/-^* male and female mice together, and genotyping was performed on all offspring using the primers and protocol provided by the Jackson Laboratory. In the C57Blk/6Jstrain, male *Bax*^-/-^ mice are infertile and female *Bax*^-/-^ mice do not produce regular litters, necessitating heterozygous matings. Frequently the SCGs in *Bax*^-/-^ mice are misshapen, tending to be elongated and thinner that the typical ovoid shape of SCG. It is clear visually that the overall volume is larger, indicative of the presence of more neurons. For EdU labeling experiments, C57BL/6J mice were used at ages P3, P5, P7, P14, and adult, as well as *Bax^-/-^* and WT (*Bax^+/-^ or Bax^+/+^*) mice at age P3. Mice used for the subtype labeling experiments were also C57BL/6J used at ages P3, P7, and P14.

### Immunolabeling

Mice were euthanized and SCG were dissected. Immediately following dissection, SCG were post fixed in 4% paraformaldehyde (Electron Microscopy Services) in 1× phosphate buffered saline (PBS) for 1 h, then cryopreserved in a 30% sucrose solution at 4°C overnight. The next day SCG were mounted and frozen in O.C.T. compound (Sakura Finertek USA) All SCG were serially sectioned at 7 µm on a cryostat (CM1950; Leica Biosystems) and collected on precleaned slides (Superplus frost, Thermo Fisher Scientific). For experiments involving GG 20 μm sections of the ganglia were used. Mice that had both GG and SCG dissected for EdU experiments were transcardially perfused with 4% paraformaldehyde in PBS before dissection. Both SCG and GG sections were washed in PBS for 10 min, and then incubated in a blocking solution containing 5% normal donkey serum (Jackson ImmunoResearch), 0.5% bovine serum albumin (Sigma-Aldrich), mouse-on-mouse blocking reagent (Vector Laboratories) and 0.3% Triton X-100 (Fisher BioReagents) in 1× PBS for 1 h at room temperature. After blocking, tissue sections were incubated with primary antibodies at their respective concentrations in the blocking solution just described at 4°C overnight. The following day sections were washed four times for 10 min each in 0.3% Triton X-100 in PBS. Tissue sections were then incubated with secondary antibodies for 2 h in the dark at room temperature. The sections were washed three times for 10 min with 0.3% Triton X-100 in PBS, then washed a final time with PBS. Slides were coverslipped using DAPI Fluoromount-G (Southern Biotechnology Associates). The following primary antibodies were used for the experiments in the SCG: Rabbit anti-CC3 (Cleaved caspase-3, 1:300, catalog no. 9661S, RRID: AB_2341188), Sheep anti-tyrosine hydroxylase (1:200, catalog no. AB1542, RRID: AB_90755), goat anti-RARRES1 (retinoic acid receptor responder 1, 1:200, catalog no. AF4657SP, RRID: AB_2284796), sheep anti-NPY (neuropeptide Y, 1:200, catalog no. AB6173, RRID: AB_305341). For immunolabeling of the GG the, we used mouse anti-TUJ1 (βIII-tubulin; 1:200, catalog no. T8578, RRID: AB_1841228) as a marker for all neurons in the ganglion. All secondary antibodies were used at 1:200 dilutions (Biotium, donkey CF488, CF543, and CF633).

### EdU injections and labeling

All reagents for injection of EdU and detection in tissue are provided in the Click-iT™ EdU Alexa Fluor™488 Imaging kit (Invitrogen, catalog no. C10337). Mice were given a single intraperitoneal injection with EdU at a dose of 8 µg/gm bodyweight. The EdU was prepared according to the manufacturer's instructions just prior to injection. After 48 h the mice were euthanized, and the SCG and GG were dissected and sectioned as previously described. Both SCG and GG sections were co-labeled for EdU and their respective cell markers. Just prior to beginning immunolabeling, reagents used in the Click-iT™ Reaction Cocktail were prepared. SCG and GG sections were washed once with PBS for 10 min, once with 3% bovine serum albumin (Sigma-Aldrich) in PBS for 10 min, and then once with 0.3% Triton X-100 in PBS. Following these washes the slides were washed again twice for 10 min each using with 3% bovine serum albumin in PBS. While sections were incubating in BSA, the Click-iT™ Reaction Cocktail was prepared with the previously made reagents, as per kit instructions. Sections were then incubated with the Click-iT™ Reaction Cocktail for 30 min in the dark at room temperature. Following incubation, sections were washed once with 3% bovine serum albumin in PBS for 10 min. Slides were then incubated with the other antibodies of interest in their normal blocking solution as previously described at 4°C overnight. Incubation with the secondary antibodies was performed as previously described. All incubation steps of the immunolabelling that occurred after EdU labelling were kept in the dark.

### Image collection

SCG Images were acquired with an SP8 Lightning confocal microscope (Leica Microsystems) using LAS-X software. All SCG and GG images were taken at 20× as z-stacks at a resolution of 1024×1024, with an optical step size of 1 μm. SCG sections that were larger than microscope field of view were captured in multiple images using the tiling function of the microscope, and maximum projection images were merged together using the “mosaic merge” feature in LAS-X. Maximum projection of the z-stacks were used for all SCG quantifications and, when applicable, merged images were used for GG quantifications. All images were collected by an observer that was naïve to the genotype and age of the tissues being imaged.

### Quantification of cleaved caspase 3+ (CC3) cells

SCGs were immunostained for CC3 and TH, and then imaged as described above. The ImageJ (NIH) cell counter tool was used to count cells in each image. Each section on the slide was counted to obtain the total number of CC3+ cells. Cells that had robust expression of CC3 and pyknotic nuclei were counted as CC3+. The sum of all sections was calculated to determine the total number of CC3+ cells in the entire ganglion. One SCG per animal was quantified.

### Quantification of TH+ and EdU+/TH+ cells

Each section of the slide was counted using the ImageJ cell counter tool to determine the total number of TH+ cells, which was robustly expressed in the cell body of nearly all neurons in the SCG. The sum of all sections was calculated to give an estimate of the total number of TH+ neurons in the SCG. For the measurement of EdU and TH double positive neurons, each section of the slide was again counted using the ImageJ cell counter tool. Cells that expressed both TH and EdU were determined, and the counts from all of the sections were summated to determine the total number of EdU+/TH+ cells in the entire ganglia. One SCG per animal was quantified.

### Quantification of EdU+/TUJ1+ cells in the GG

GG were immunolabeled for EdU and TUJ1 as described above. Each section was counted for total number of cells expressing both EdU and TUJ1, and the sum of all the sections per ganglia was calculated to determine the total number of EdU+/TUJ1+ cells in each GG.

### Statistical analyses

All statistical analyses were performed in Prism 9 software (GraphPad). Before analysis, data were evaluated for normal distribution using the Shapiro–Wilk test and were determined to be normally distributed at *P*<0.05. For all analyses we used Welch's *t*-tests and one-way or two-way ANOVAs, and significant differences were identified by Tukey post-hoc tests for ANOVAs. All graphs and error bars shown represent the mean± standard error of mean (s.e.m.), and the indicated extent of significance is *P<0.05, **P<0.01, ***P<0.001, ****P<0.0001.

## Supplementary Material

10.1242/biolopen.060541_sup1 Supplementary information
