# Supplementary material for: Identification of a postnatal period of interdependent neurogenesis and apoptosis in peripheral neurons
Source: Biol Open. 2024 Nov 11;13(11):bio060541. doi: 10.1242/bio.060541 (PMC11583921; doi:10.1242/bio.060541)
Supplement: Supplementary information [file biolopen-13-060541-s1.pdf]

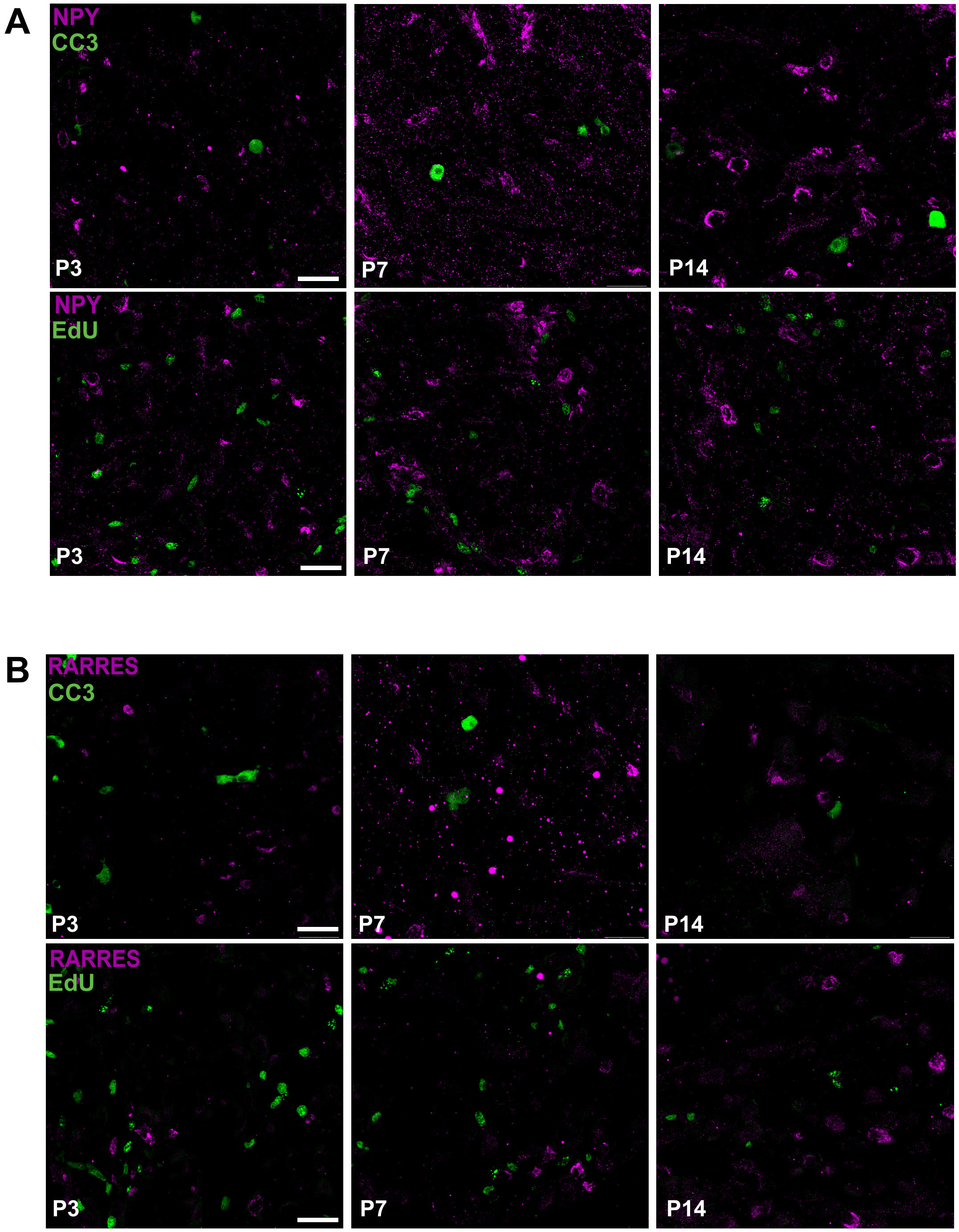

Fig. S1. Postnatal apoptosis and neurogenesis does not occur in the Neuropeptide Y-expressing (NPY) or RARRES-expressing subpopulations in the SCG.

Biology Open • Supplementary information

Mice were injected with EdU at the age indicated on the panels (P3-P14) and after 48 hours the SCGs were isolated. (A) Immunofluorescence images of C57/Blk6j mice labeled with NPY (magenta) and CC3 (green) are shown in the top panels, and images of SCGs labeled for EdU (green) and labeled for NPY (magenta) are displayed in the bottom panels. (B) Immunolabeling was performed on mice 48 hours after EdU injection at P3, P7 and P14 (labeled in each panel) and analyzed with immunofluorescence labeling of CC3 (green) and RARRES (magenta) in the top panels, or EdU (green) and RARRES (magenta) in the bottom panels. Scale bar = 75 μm and n=3-5 for all conditions. Note that NPY and RARRES expression was more apparent at P7 and P14, suggesting that these populations don't begin to emerge until postnatal age.
